# Behavior, Color Change and Time for Sexual Inversion in the Protogynous Grouper (*Epinephelus adscensionis*)

**DOI:** 10.1371/journal.pone.0019576

**Published:** 2011-05-25

**Authors:** Richard J. Kline, Izhar A. Khan, G. Joan Holt

**Affiliations:** 1 Marine Science Institute, The University of Texas at Austin, Port Aransas, Texas, United States of America; 2 Dexter National Fish Hatchery and Technology Center, United States Fish and Wildlife Service, Dexter, New Mexico, United States of America; University of Sussex, United Kingdom

## Abstract

Hermaphroditism, associated with territoriality and dominance behavior, is common in the marine environment. While male sex-specific coloration patterns have been documented in groupers, particularly during the spawning season, few data regarding social structure and the context for these color displays are available. In the present study, we define the social structure and male typical behavior of rock hind (*Epinephelus adscensionis*) in the wild. In addition, we detail the captive conditions and time period necessary to induce the onset of the sex-specific coloration and sexual change. At six oil production platform locations in the Gulf of Mexico, rock hind social group size and typical male rock hind social behavior were documented. We observed a rapid temporary color display in rock hind that could be turned on and off within three seconds and was used for confronting territory intruders and displays of aggression towards females. The male-specific “tuxedo” pattern consists of a bright yellow tail, a body with alternating dark brown and white patches and a dark bar extending from the upper mandible to the operculum. Identification and size ranges of male, female and intersex fish collected from oil platforms were determined in conjunction with gonadal histology. Rock hind social order is haremic with one dominant male defending a territory and a linear dominance hierarchy among individuals. In five captive experiments, the largest remaining female rock hind displayed the male specific color pattern within 32d after dominant male removal from the social group. To our knowledge, this is the first evidence in a grouper species of color patterning used to display territoriality and dominance outside of spawning aggregations. The behavioral paradigm described here is a key advance that will enable mechanistic studies of this complex sex change process.

## Introduction

Hermaphroditism, associated with territoriality and dominance behavior are common in the marine environment, especially with reef or substrate-associated fish such as groupers and basslets (Serranidae), wrasses (Labridae), and parrotfishes (Scaridae). Dominance hierarchies have been described in several species and are common in protogynous hermaphrodites that change sex from female to male, often resulting in the largest fish in a social group being a dominant and highly aggressive male [1], [2], [3].

Sex-specific coloration patterns are present in many vertebrate classes including mammals [4], birds [5], amphibians [6] and coloration differences in the dominant male have been documented in many fish species [1], [2], [3], [7], [8], [9], [10]. These patterns are particularly evident in protogynous species such as parrotfishes, wrasses [2], [11], [12] and groupers (Serranidae, *Epinephelinae*) [13], [14]. Temporary and ephemeral coloration patterns have been noted in both marine and freshwater fish species, especially during the spawning season and for agonistic encounters between males [15]. While differing coloration patterns exist, changes in head coloration body contrast patches and fin edges are common locations of temporary and ephemeral coloration [15].

Wrasses have been extensively studied in terms of behavior and protogynous sex change [3], [7], [12], [16] and the dominance hierarchy has been well described [2]. Typically a single dominant male with a permanent distinctive color pattern controls a harem of females that can greatly vary in number depending on population size and available reef habitat. Aggression from the dominant male is typically directed at territorial intruders and the largest female in the harem. This is presumed to occur because the largest female fish is almost always the next in line to change sex [3], [7], [17], [18], [19].

Several species of grouper are documented to display distinctive male coloration patterns during spawning aggregations including the red hind (*Epinephelus guttatus*), rock hind (*E. adscensionis*), scamp (*Mycteroperca phenax*), gag (*M. microlepis*), coney (*Cephalopholis fulva*), tiger (*M. tigris)*, and black (*M. bonaci*) groupers [14], [20]. In contrast to ephemeral coloration patterns, these coloration patterns persist even after capture and hours after death in some species [13], [14]. In a few grouper species, ephemeral coloration patterns have been noted but these are only documented very near the time of gamete release [8], [13], [20]. Blue head wrasses also exhibit variations in color patterning in males in terms of permanent pigmentation patterns as well as short term variation between “opalescent” spawning coloration and deeper hued coloration patterned associated with territorial defense [21] but similar data are not available for the rock hind.

An excellent method to study these behavioral changes in protogynous fishes has been through male removal experiments [2], [3], [16], [22]. These experiments have been conducted in field and laboratory settings to determine the conditions and time duration to induce sex change in several protogynous species. Typically, two major changes occur during these experiments: 1) the onset of male-like behavior and aggression; and 2) transition of the gonad from ovary to testis. In the wrasses and gobies (Gobiidae), behavioral sex change is rapid and can occur within minutes to hours of dominant male removal in the largest remaining fish [2], [23]. Gonadal sex change takes considerably longer than behavioral change due to the need to reorganize the gonad morphologically and in terms of steroidogenic capacity. In bluehead wrasse, this process takes as few as eight days [2]. In grouper species, gonadal transition from ovary to testis occurs within three weeks in the half-moon grouper (*Epinephelus rivulatus*) [22] and less than seven weeks in the chocolate hind (*Cephalopholis boenak*) [19]. However, information on behavioral changes concurrent with gonadal changes is lacking in any grouper species as demonstrated previously in the bluehead wrasse [2] and Anthias (*Anthias squamipinnis*) [24].

The focus of this study is the rock hind, a protogynous grouper that has a wide geographic distribution from Ascension Island in the Eastern Atlantic to North Carolina in the Western Atlantic and throughout the Caribbean and the Gulf of Mexico [25]. The rock hind was selected as a behavioral model in this study due to the lack of behavioral data and the context of color patterning in groupers (Epinephelinidae). Moreover, the rock hind is relatively abundant on oil and gas production platforms in the Gulf of Mexico and is observed at depths accessible by SCUBA divers [26], [27]. In a brief description concerning spawning aggregations of several grouper species by Colin [14], rock hind males were reported to be sexually dichromatic, having black and white blotches in the context of spawning and courtship.

Here we present behavioral observations of the social structure and dramatic, temporary sex-specific color displays in wild rock hind. Furthermore, we present experimental data on the conditions and time period necessary to induce the onset of the sex-specific coloration and sex change in captive experiments.

## Methods

**Figure 1:**
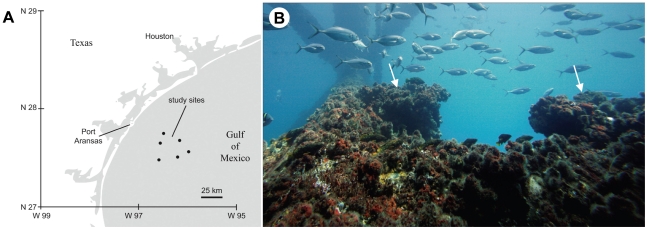
Locations and characteristics of rock hind habitat. A) Map of Texas coast showing oil platform study and sampling sites (black dots). B) Picture of oil platform support rung with heavy barnacle growth showing two adjacent rock hind territories (white arrows).

Field observations of rock hind social organization and color patterning were made on SCUBA at depths from 6–15 m on oil and gas production platform habitats in the Gulf of Mexico near Port Aransas, Texas (Fig. 1A). Production platforms in the Gulf of Mexico have been in place for 20 years or more [27] and typically have considerable overgrowth of encrusting organisms such as barnacles, sponges and corals, as well as abundant fish populations [27] (Fig. 1B). Collections for sex identification and captive study of rock hind behavior were also made on platforms in the Gulf of Mexico on SCUBA using spear fishing and underwater hook and line fishing. For captive experiments, fish were transported to facilities at the University of Texas at Austin Marine Science Institute, Port Aransas, TX USA. Field observations of rock hind social behavior were made on 20 social groups at six platform locations in the Gulf of Mexico from March to November 2008. Rock hind behavior was observed by divers on SCUBA and from supplemental underwater videos. Assessments of rock hind social group size were recorded along with coloration patterns and distance between territories. After observations, rock hind that were previously observed in male-typical display as well as other fish in the vicinity of each social group were collected for sex determination by spear fishing and underwater hook and line fishing. Due to the ephemeral patterning of male rock hind and flight during capture attempts, the first effort was made to collect displaying male fish from each location. Aside from displaying males, not all fish observed were collected and potentially others not quantified in observations could have been collected. On the boat, rock hind were anesthetized with an overdose of clove oil (0.1 ml/l) and measured for total length. Fish were killed by severing the spinal column and gonadal tissue was removed and preserved in 10% buffered formalin until further processing.

Gonadal tissue was rinsed then cryoprotected in 30% sucrose solution followed by embedding in mounting media (OCT Compound, Sakura, Torrance, CA). Fifteen micron cross-sections were made on a cryostat and thaw mounted on slides (Superfrost plus, Erie Scientific). Sections were air dried then rehydrated in PBS (20 mM phosphate base, 150 mM NaCl, pH 7.6) and stained using Myers' hematoxylin and eosin. After staining, sections were dehydrated in an alcohol series and cleared with xylene. Slides were cover-slipped with Cytoseal-60 (Richard-Allan, Kalamazoo, MI) and allowed to dry overnight. Representative sections were imaged on a Nikon eclipse microscope with black and white high resolution camera (CoolSnap cf mono). Sex determined from gonadal sections was compared to behavior and color patterning recorded in focal fish before capture and to size recorded after capture.

### Rock hind behavioral manipulation experiments

Thirty rock hind were captured from oil platform locations in April and May for use in captive experiments to determine the time course of behavioral and occurrence of gonadal sex change. Fish were placed in a dechlorinated fresh water bath for 2 minutes to remove ectoparasites [28], then held together in a 3 m diameter tank for one week to recover from capture and transport. Captive conditions were 26°C temperature and 12.5∶11.5 light∶dark photoperiod for all experiments. Fish were fed a mixture of chopped sardines, shrimp and squid to satiation every day. After the recovery period, fish were anesthetized and examined for sexual condition by applying pressure on the ventral side to collect milt or by inserting a small cannula into the oviduct to collect a biopsy sample. Biopsy samples were observed under the microscope following the methods of Neidig [29].

Fish were divided among five tanks in which one male, confirmed by expressing milt (290–320 mm), one medium sized female (260–280 mm), and three smaller females (180–250 mm), were placed into each group (confirmed from biopsy samples). Fish in which the sex could not be definitively determined were excluded from the experiments. A single habitat structure made of three 0.5 m sections of 10 cm diameter PVC pipe stacked in a pyramid configuration and held together with plastic cable ties was placed into each 1.2 m depth×3 m diameter tank. Five rock hind social groups with identical habitats were set up for the experiments.

Three weeks were allowed for the development of social hierarchies; the characteristic color display in each male (determined in the field studies) was verified before any behavioral manipulations were made. After this, the male from each tank was removed. Daily visual and video observations of the focal fish (the largest female) were made for seven weeks for 1.5 h near the end of each day when the probability of observing male behavior was highest according to preliminary observations. Observations were scored for onset of aggression, behavioral changes and the appearance of the characteristic male color pattern. Data from the five replicated experiments were compared for major changes in behavior and onset of male typical behavior profiles. Mean and standard error were calculated for time from male removal to: first observation of aggression in the largest remaining fish, first change in coloration and onset of full color display by the dominant fish. A timeline for rock hind behavioral sex change was constructed using the data from five replicated groups. Data is reported as mean ± S.E unless otherwise noted. This study was approved by the University of Texas Institutional Animal Care and Use Committee (Protocol #04052403).

## Results

### Field observations

**Figure 2:**
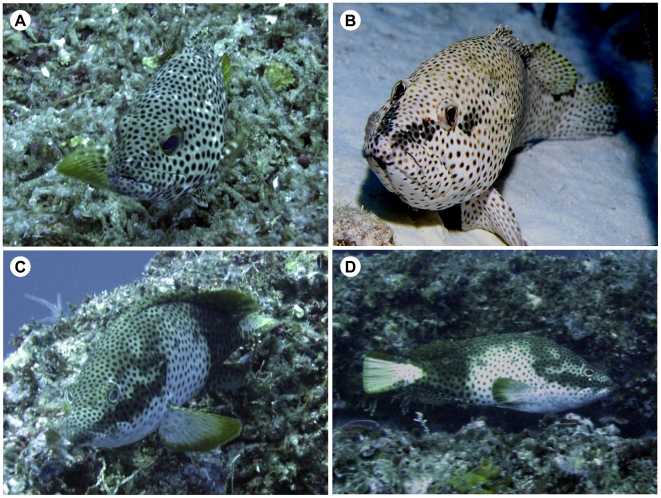
Photographs of typical rock hind coloration patterns. Head markings of rock hind A) male or female cryptic pattern; B) male or female aggressive eye-bar pattern; C) male-specific territorial “tuxedo” pattern; and D) Aspects of the “tuxedo” pattern showing white and brown patches, yellow tail with brown border and head markings. The “tuxedo” pattern is an ephemeral color pattern. Males can switch from any of the patterns (A, B or C) very quickly.

The typical color pattern of both male and female rock hinds is a cryptic camouflage pattern consisting of white, light brown and red-brown spots (Figure 2A). When agitated, both male and female rock hind can display a darkened horizontal bar extending from the upper mandible through the eye and ending at the operculum (Fig. 2B). However, the male also displays a dramatic, temporary color pattern that we designated the “tuxedo” pattern (Figure 2C–D) consisting of a bright yellow tail, and a body with alternating dark brown and white patches. In addition, a distinctive pattern was displayed on the head consisting of a dark bar extending from the upper mandible to the operculum and a bright white spot below the eye (Fig. 2D). Although not specifically measured, this pattern appeared to be very consistent from fish to fish in field observations.

Male rock hind were observed guarding territories during all months studied, and this tuxedo pattern was displayed when confronting potential territory intruders. It was also observed when males were displaying aggression towards the females in the harem, especially the largest ones. This color change from camouflage to tuxedo pattern was quite fast and could be turned “on” or “off” within 3 seconds. Accompanying the tuxedo pattern was a characteristic “head shake”, a lateral side to side movement of 3–5 seconds that was directed towards females within the harem or to territorial intruders (Movie S1).

### Rock hind social behavior

Rock hind social order was observed to be haremic with one dominant male defending a territory and several smaller females residing within that territory. A linear dominance hierarchy was observed in many cases with one or two larger female(s) holding sub-territories and displaying aggressive behavior (chasing, biting and eye-bar display) to smaller females.

Rock hind behavior and harem size appeared to depend on the size and quality of the available habitat in the field. For example, on offshore pinnacle reefs approximately 6 m in diameter, a single territory and harem was observed on each mound with 10 m of vertical relief (R. Kline, pers obs). In contrast, production platforms had a three dimensional lattice of structures with support rungs spaced every 10 m of depth, and several social groups could be observed on each rung if sufficient barnacle or sponge growth was present. Territory spacing was also dependant on habitat composition and typical spacing ranged 6 to 10 m; however with sufficient structure, only 3 m of spacing between territories was observed at one site.

### Sex ratio

Rock hind social group size observed in twenty social groups over six sites ranged from five to nine individuals with a mean of 7.4±0.3 individuals, based on 30 minute observations of each group and proximity to defined territory of the displaying male near a specific hole or crevice that the focal male occupied. This would equate to a mean sex ratio of 6.4 females to one male in the six locations observed. Solitary tuxedo displaying males were observed at three sites. These fish occupied small areas with little structure and no subordinate fish present. Occasionally they were observed roving into territories and were chased away by the dominant male of that territory.

### Gonadal histology

**Figure 3:**
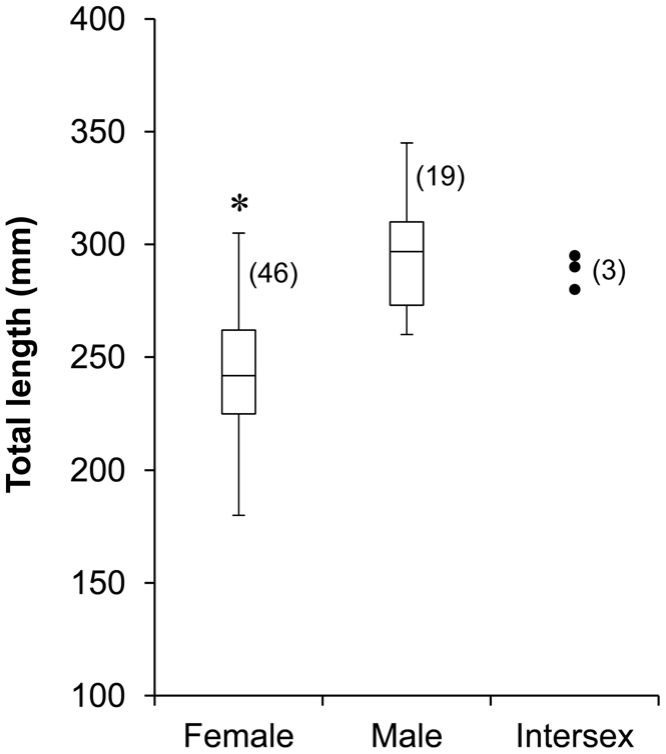
Length-at-sex plots. Box and whisker plots of female and male rock hind total length with data points for intersex fish captured at 6 oil platform study sites in the Gulf of Mexico during March through November 2008. Asterisk (*) indicates mean female total length was significantly different from male and intersex mean total length (ANOVA with Tukey comparison p<0.001 and p = 0.009 for F vs. M and F vs. intersex, respectively). Sample size is indicated in parentheses.

Of the 68 rock hind sampled from platforms in the Gulf of Mexico, 46 were female with mean TL 240±4 mm; 19 were identified as male with mean TL 297±6 mm and 3 fish were intersex with mean TL 288±4 mm. Mean male and intersex TL was significantly different from female TL (ANOVA with Tukey comparison p<0.001 and p = 0.009 for F vs. M and F vs. intersex, respectively). A size at length diagram is presented in Figure 3, demonstrating the size ranges observed in sampled fish. Overlap was observed between males and females at 260 to 305 mm, and no males were captured below this size range. Mature female rock hind were observed ranging in size from 180 mm to 305 mm.

**Figure 4:**
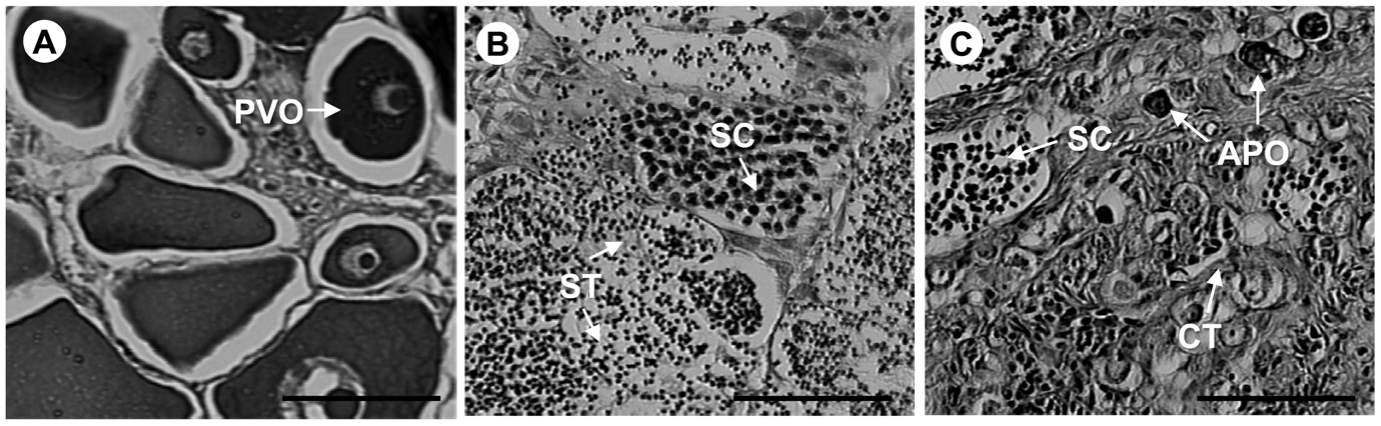
Gonadal tissue cross-sections. Representative cross sections of A) female, B) male, and C) intersex gonadal tissue from rock hind captured on oil platform habitats in the Gulf of Mexico. PVO: previtellogenic oocytes; SC: spermatocytes; ST: spermatids; APO: atretic previtellogenic oocytes; and CT: connective tissue. Scale bars = 50 µm.

The gonadal tissue from 10 fish captured while displaying the tuxedo pattern confirmed that they were male. All had well developed testes and no evidence of ovarian tissue; although in three cases yellow-brown bodies were noted in gonadal sections, potentially indicating remnants of atretic oocytes and recent sex change [30]. Due to the ephemeral nature of color patterning in rock hind, some fish used in this study were captured at each site with no behavioral context. Males were also observed in several stages of development, with the majority of testes having spermatocytes and spermatids present (Fig. 4B). Females were observed in several stages of ovarian development from previtellogenic to late stage atretic oocytes, with the most common being previtellogenic oocytes (Fig. 4A), however no females were captured with ripe ovaries, indicating that they were captured outside the spawning period.

Three intersex fish were identified in the field collections. On gross examination, these fish had reduced gonad size and the gonads appeared dark in color compared to typical ovaries and testes. In cross-section, all three intersex fish had atretic previtellogenic oocytes, dense connective tissue and testicular tissue in the early stages of development, with numerous crypts of spermatocytes and few crypts of spermatids (Fig. 4C).

### Behavioral experiments with captive fish

Before male removal, observations revealed the presence of a single dominant male that would occupy the tube structure and periodically rush at females that had entered the structure causing them to flee. This behavior was repeated many times during each observation. In all social groups the largest female also had a defined territory on one side of the structure near an opening and would prevent entry of any of the smaller females into this space. The male would “patrol” and occasionally flash the tuxedo pattern while swimming around the entire tank. During these patrols the male would sometimes displace the largest female from her territory and occupy it or in some cases, physically push the female out of the way with his snout. This behavior was most often observed during the last hours of light and was prevalent throughout the day before spawning in experiments as described below.

**Figure 5:**
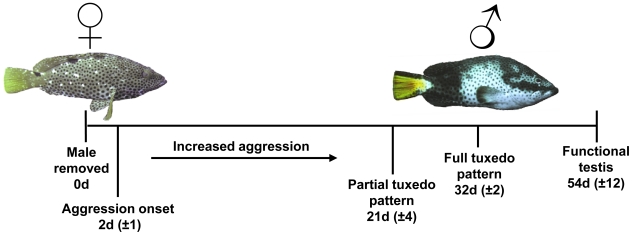
Timeline for sex change in rock hind. Timeline in days ±1 S.E. for behavior and color patterning of the largest female after male removal based on replicated captive observation experiments (n = 5). Each replicate had one habitat structure and starting conditions of one male, one intermediate and three smaller rock hind in each 3 m diameter tank.

A timeline for rock hind behavioral change after male removal is shown in Figure 5. In the male removal experiments, the remaining fish would typically hide for one day post male removal and little activity was observed during this period. At day two, aggression by the largest female towards the smaller fish was observed and the ±1 d qualitative intensity of these interactions increased gradually over several weeks. Increased patrolling of the entire tank and a partial tuxedo display was observed by 21±4 d in the largest female. The partial display showed the tuxedo body coloration and yellow tail but no prominent dark bar below the eye. The timing of the onset of a complete tuxedo display was remarkably consistent occurring at 32±2 d in all 5 groups.

In two of the groups, the recently sex-changed fish successfully fertilized eggs at 42 and 67 d respectively. Fertilization was verified by following the egg development over 24 hours. After spawning, females had numerous bite marks on both flanks, apparently from the recently sex-changed male. Fish from this experiment were used in other studies and were not checked for gonadal state immediately after the experiment. However, all of the largest fish from the three groups that did not spawn were verified as males by applying pressure to the abdomen and expressing milt. All remaining fish from the experiment were later confirmed to be females by gonadal biopsy.

## Discussion

Sex-specific color patterning, observed in many reef-associated species during the spawning season, is usually reported as permanent, remaining present for hours to days after death in the bluehead wrasse [16] and groupers [14]. However, the intricate and striking tuxedo pattern reported here in rock hind is temporary, can be turned on and off very quickly, and is not present after capture or after death. Ephemeral color patterning has been noted in other species for use of agonistic encounters and spawning behavior [15] but to our knowledge, this is the first evidence of color patterning in a grouper species used for display of territorial defense and dominance except during spawning aggregations. The rapid change in color patterning reported here is likely under neural control as reported in cichlid (*Astatotilapia burtoni*) [31].

The behavior and social structure observed in rock hind on offshore platforms is similar to that reported for the half-moon grouper on reefs in Australia [22]. The male to female sex ratio of 1∶6.4 found in this study is also comparable to that seen in half-moon grouper (1∶6). In addition, Mackie [22] reported the presence of bachelor males at the reef study site similar to that reported here. Although he described spermatogenic tissue within the ovaries of many females, this was not observed in rock hind ovaries in this study; however Mackie sampled fish during the spawning season when many females had well-developed ovaries. In the present study, none of the ovaries examined were well developed and the fish were apparently collected outside of the spawning season.

Rock hind displayed a linear social hierarchy similar to that reported in cleaner wrasse [3]. In the field and laboratory experiments, rock hind males were most aggressive toward the largest females that held sub-territories and larger females were most aggressive toward small females. This selective aggression by the male has been presumed by Robertson [3] in wrasse because the largest female is next in line to change sex. This behavior in rock hind was highlighted in some preliminary experiments with mirrors whereupon seeing a reflection, rock hind would attempt to chase a similar-sized image even when other sized fish were present in the reflection (R. Kline, unpublished data).

No male or intersex rock hind were observed below the size of 260 mm total length in the field collections, suggesting that rock hind have a minimum size required to transition to male. In some hermaphroditic species, a minimum size threshold for sex change has been proposed (e.g. Shapiro [32]); however, the determination of minimum size for rock hind sex change would require a much larger field sample size. Behavioral experiments in the present study were designed to avoid this issue by selecting fish within the male/female overlap range.

In fishery studies of other grouper species across multiple habitat types, a wide range of male sizes is reported [19], [22]. In some grouper species, primary males are documented that directly mature as males and never function as the females [19], [33]. The data for size at sex in the present study provide no evidence of primary males in rock hind.

Direct determination of sex by examination of biopsy samples (this study; also see Mackie 2003) or reliable secondary sex characteristics [19] is advised in behavioral experiments with small groupers because individuals captured from several sites cannot be assigned a sex identification by relative size alone. In general, we observed that rock hind males were larger than females among neighboring territories. However, even at greater depths at the same site and from site to site, females could be larger than some males captured at other locations.

Although the gonadal state of males at the onset of color pattering in behavioral experiments was not investigated here, participation in spawning activity by two fish in captivity within weeks after they first displayed the tuxedo pattern, provides compelling evidence that these fish were at least intersex if not fully sex-changed males by 32±2 d. Furthermore, the field data support this by the fact that all tuxedo-displaying males sampled from the field were identified as male by gonadal histology. Other studies in grouper show that the half-moon grouper transition to producing mature sperm by three weeks in many cases [22] and less than seven weeks in the chocolate hind [19]. Interestingly, the time required for induction of sex change with steroid implants in groupers [34], [35], [36] is similar to that reported here for behavioral experiments, suggesting that the change in steroid profiles are initiated quickly after male removal in order to transition the gonad within this time period.

Aside from the satellite males noted, only one male was observed within each social group at the platform sites. Males that function as parasitic spawners have been described in numerous fish species [37]. Sneaker males that look and behave like females within the social group have been described in haremic and lek-based mating systems in the gonochoristic midshipman (*Porichthys notatus*) [38], and protogynous bluehead wrasse [39]. However sneaker males have not been described in groupers and were not observed in this study. In the tiger grouper, smaller streaker males rush in and release gametes during pair spawning of a larger male and a female [13]. It is possible that rock hind satellite males function as parasitic spawners as well, and in this scenario it would be beneficial to remain male and streak in to spawn or take over a recently vacant territory rather than change back to female. It is unclear if rock hind can change back from male to female as described for the chocolate hind [19]. Although this condition might be artificially induced, the presence of satellite males occupying sub-optimal habitat and the relatively long period of sex change reported here suggest that it is unlikely to occur in the wild rock hind populations observed in this study.

In captive experiments we used fish that were likely unfamiliar with one another to create social groups. In the wild this is not likely to be the case as many of the group would be familiar after the male was removed or displaced. The effects of this long term familiarity on social group dynamic are presently unknown. However we did keep the groups together for three weeks before manipulation and in all cases when unfamiliar individuals were forced together in a captive group, the largest individual, a male assumed the role at the top of the hierarchy.

Although solitary “tuxedo” displaying males were seen in wild rock hind populations, their presence was intentionally avoided in the captive sex change studies reported here. In wild populations, these solitary males likely play an important role in the social dynamics of rock hind harems when the resident dominant male is lost. Territory-holding half-moon grouper males removed from reefs by Mackie [22] in Australia were replaced by solitary immigrant males in 45% of the cases. Curiously, after the immigrant males were removed in Mackie's study, smaller remaining fish did not move in to replace them, suggesting a minimum size is necessary. The importance of these solitary males and how it affects the sex change process in the largest remaining female in wild populations of rock hind warrants further study.

Color displays, especially the dark band below the eye in the tuxedo pattern, appear very important to the male display. The presence of an eye-bar has been extensively studied in the gonochoristic cichlid, and it is only displayed in dominant males [40]. In rock hind, both sexes can display a bar through the eye. However, the male specific tuxedo pattern appears to be a chromatically opposite pattern to the aggressive eye-bar pattern that both males and females can display (see Figure 2B vs. 2C). When captive rock hind males are shown the tuxedo pattern on a video monitor, they react by aggressive approach and reciprocal display of the tuxedo pattern or eye bar, which is not seen towards images without the tuxedo bar on the face (R. Kline, unpublished data). It is also possible that other components of this territorial visual display are not visible to the human eye and occur in the UV spectrum as seen in the damselfish (*Pomacentrus amboinensis*) [41].

### Conclusions

Rock hind is a protogynous hermaphrodite that uses a striking and temporary “tuxedo” display in association with aggression and dominance and defense of territories. In behavioral experiments, female rock hind displayed the male specific “tuxedo” color pattern within 32 d of dominant male removal from the social group. This is the first evidence reported for groupers of temporary color patterning used for display of territoriality and aggression outside of the spawning season. A key result of this study is the establishment of an effective experimental paradigm for inducing sex change under captive, controlled conditions and that is an important advance enabling mechanistic studies of the complex sex change process.

## Supporting Information

Movie S1 **Rock hind male behavior.** Typical rock hind *Epinephelus adscensionis* male patrolling behavior, “head shake” and tuxedo pattern display.
(MP4)
